# Comparative analysis of Robotic-Assisted, Laparoscopic, and open radical nephrectomy: Utilization, Costs, and clinical outcomes

**DOI:** 10.1007/s11701-025-02995-x

**Published:** 2025-11-24

**Authors:** Daniel Y. Huang, Costas D. Lallas, Raegan M. Davis, Scott W. Keith, Patrick J. Moeller, Inkyu K. Kim, Anushka Ghosh, Francisco Aguirre, Rasheed A. M. Thompson, Emmanuel F. Drabo, Vittorio Maio

**Affiliations:** 1 College of Population Health, Thomas Jefferson University, Philadelphia, PA USA; 2 Department of Urology, Sidney Kimmel Medical College, Thomas Jefferson University, Philadelphia, PA USA; 3 Division of Biostatistics and Bioinformatics, Sidney Kimmel Medical College, Thomas Jefferson University, Philadelphia, PA USA; 4 Sidney Kimmel Comprehensive Cancer Center, Thomas Jefferson University, Philadelphia, PA USA; 5 Department of Health Policy and Management, Johns Hopkins University Bloomberg School of Public Health, Baltimore, MD USA; 6 Asano-Gonnella Center for Research in Medical Education and Health Care, Sidney Kimmel Medical College, Thomas Jefferson University, Philadelphia, PA USA; 7 College of Population Health, Thomas Jefferson University, 901 Walnut St., 10th Floor, Philadelphia, PA USA

**Keywords:** Comparative analysis, Laparoscopic radical nephrectomy, Open radical nephrectomy, Robotic-assisted radical nephrectomy, Renal cancer

## Abstract

**Background:**

Minimally invasive approaches, including laparoscopic (LARN) and robotic-assisted radical nephrectomy (RARN), have gained adoption over open surgery (ORN) for renal cancer, despite RARN’s higher costs. This contemporary study evaluates trends in RARN, LARN, and ORN use and compares their hospital costs, clinical complications, and mortality rates.

**Methods:**

Patients undergoing radical nephrectomy (2016–2019) were identified from the National Inpatient Sample (NIS). Procedures were classified as RARN, LARN, or ORN using ICD-10 and Procedure Coding System codes. Patient demographics and comorbidities, hospital characteristics, length of stay (LOS), clinical complications, and hospital costs were analyzed. Trends in utilization were assessed, and regression models adjusted for patient and hospital factors examined associations between surgical approach and inpatient perioperative outcomes, including complications, mortality, LOS, and hospital costs.

**Results:**

Among 154,115 patients, 39.5% underwent LARN, 25.7% RARN, and 34.8% ORN. Annual RARN utilization increased (21.8% to 29.6%), while LARN declined (44.8% to 35.2%). RARN was more common in older and comorbid patients. Median costs were lowest for LARN ($13,950) compared to RARN ($16,771) and ORN ($17,821). Both RARN and LARN had lower inpatient perioperative complications, blood transfusion rates, and mortality than ORN. RARN and LARN were associated with reduced LOS and costs relative to ORN. While RARN was 15% more expensive than LARN, it had 5% shorter LOS. A limitation was the absence of tumor characteristic data.

**Conclusions:**

RARN and LARN are increasingly used and both demonstrated better inpatient perioperative outcomes than ORN. However, RARN offers no clear clinical advantage over LARN and remains more costly than LARN.

**Supplementary Information:**

The online version contains supplementary material available at 10.1007/s11701-025-02995-x.

## Background

Renal cell carcinoma (RCC) is a significant public health concern, accounting for approximately 4.2% of adult malignancies globally and over 74,000 new cases each year in the United States [1]. The standard of care of RCC often involves radical nephrectomy, which remains the curative treatment of choice for large tumors and the preferred approach for T1 and T2 tumors that are not candidates for nephron-sparing surgery [2]. However, the advent of minimally invasive surgery has led to a shift towards techniques such as laparoscopic radical nephrectomy (LARN) and, more recently, robotic-assisted radical nephrectomy (RARN) [3–6].

While LARN offers advantages such as shorter recovery times and lower postoperative morbidity, RARN has emerged as a refinement of this approach, providing enhanced precision and visualization through robotic articulation and advanced imaging capabilities. Despite its potential clinical advantages, RARN is more costly, prompting ongoing debate regarding its cost-effectiveness relative to LARN [3, 6, 7]. Current evidence on the comparative benefits of RARN, particularly regarding clinical outcomes and economic impact, remains limited and is largely derived from single-institution studies [6, 8, 9].

This study aims to provide a contemporary comparative analysis of RARN, LARN, and open radical nephrectomy (ORN) in terms of utilization, costs, and clinical outcomes in a large cohort of renal cancer patients.

## Materials and methods

### Data source and study design

This retrospective cohort study utilized data from the 2016–2019 National Inpatient Sample (NIS), the largest publicly available all-payer inpatient healthcare database in the United States [10]. The NIS, maintained by the Healthcare Cost and Utilization Project (HCUP) under the Agency for Healthcare Research and Quality (AHRQ), captures a 20% stratified sample of hospital discharges and applies survey weights to generate national estimates. Given its comprehensive scope, the NIS enables robust, national-level analyses of trends, providing a valuable source for assessing the impact of surgical approach on renal cancer management. The NIS database has been extensively used to estimate hospital costs associated with both urological and non-urological major surgeries [11–15].

### Study population

Patients aged 18 years or older with a diagnosis of renal cancer who underwent radical nephrectomy were identified using the International Classification of Diseases (ICD)−10 Clinical Modification (CM) and Procedure Coding System (PCS) codes specific to LARN (0TT04ZZ, 0TT14ZZ, 0TT24ZZ) and ORN (0TT00ZZ, 0TT10ZZ, 0TT20ZZ). RARN procedures were distinguished from LARN by the presence of an additional PCS code for robotic assistance with a procedure of the trunk region (8E0W8CZ, 8E0W0CZ, 8E0W3CZ, 8E0W4CZ, 8E0W7CZ, 8E0WXCZ).

### Demographic and clinical characteristics

Patient-level characteristics included age, sex, race, primary payer, and median household income quartile (based on Zip code) as provided by the NIS database. Patient comorbidities were assessed using the Elixhauser Comorbidity Index (ECI) from HCUP [16]. Hospital-related factors included facility type, region, and bed size. Hospital surgical volume was categorized as low (5 or fewer procedures), intermediate (6 to 24), or high (25 or more) over the study period to minimize cell counts below 11, in compliance with NIS data suppression requirements. All demographic characteristics were weighted according to HCUP discharge-level estimates.

### Outcomes

ICD-10-CM diagnosis and PCS codes were utilized to identify inpatient blood transfusions and complications, including cardiac, respiratory, genitourinary, vascular, wound-related, bleeding, and other medical or surgical complications (Supplemental Table 1). Outcomes specific to the NIS dataset included in-hospital mortality, hospital length of stay (LOS), discharge disposition (routine discharge to home vs. other settings such as rehabilitation or skilled nursing facilities), and total hospitalization costs. Cost estimates were calculated by applying the HCUP cost-to-charge ratio to total charges. Cost estimates below $2,000 (60 procedures) were removed from the analysis.

### Statistical analysis

Descriptive statistics were calculated for all variables of interest. Multivariable regression models, adjusted for demographic and hospital factors, were used to compare outcomes across surgical approaches. The following patient characteristics were included in the models as dichotomous variables: sex, age (≤50 vs. >50), race (White vs. non-White), Zip code median income (Q4 vs. Q1-Q3), and primary payer status (private insurance vs. non-private payer). Patient comorbidity (ECI) was included as a count variable. Hospital characteristics included in the models as dichotomous variables were hospital type (urban teaching vs. other), region (South/Midwest/West, North), and surgical volume (high vs. low/intermediate). Logistic regression was employed for binary outcomes, including overall and specific complications, non-routine discharge, and mortality. Models for vascular, bleeding, and miscellaneous medical complications were not fitted due to the low number of events. Negative binomial regression was used to account for overdispersion in LOS count data, while Poisson regression was employed to model complication counts [17]. Linear regression was applied to cost data that were log-transformed to normalize their distribution. In a secondary analysis, the same regression models were used to compare RARN and LARN across all outcomes. A significance level of α = 0.05 was applied. By incorporating the NIS sampling design variables, all analyses were adjusted for sample weights, stratification, and clustering by hospital. Statistical analyses were conducted using SAS v9.4 (SAS Institute, Cary NC).

## Results

A sample-weighted national estimate of 154,115 patients underwent radical nephrectomy from 2016 to 2019, among whom 39.5% received LARN, 25.7% RARN, and 34.8% ORN (Table 1). During this period, the annual use of RARN increased from 21.8% to 29.6%, and LARN declined from 44.8% to 35.2%. ORN remained consistently performed, accounting for approximately one-third of cases annually (Supplemental Fig. 1).

**Table 1:** Characteristics of patients undergoing radical nephrectomy by surgical technique

	LARN	RARN	ORN
Patients, N (%)	60,855 (39.5)	39,590 (25.7)	53,670 (34.8)
Age, Mean ± SD	57.6 (16.7)	62.9 (14.9)	57.9 (18.2)
Age, N (%)			
≤ 50	18,250 (30.0)	7,000 (17.7)	15,470 (28.8)
51–60	12,940 (21.3)	7,705 (19.5)	11,930 (22.2)
61–70	15,525 (25.5)	11,725 (29.6)	13,825 (25.8)
≥ 71	14,140 (23.2)	13,160 (33.2)	12,445 (23.2)
Sex, N (%)			
Female	28,135 (46.2)	16,510 (41.7)	22,585 (42.1)
Male	32,685 (53.7)	23,060 (58.2)	31,065 (57.9)
Missing	35 (0.1)	20 (0.1)	20 (0)
Race, N (%)			
White	40,565 (66.7%)	29,205 (73.8%)	35,905 (66.9%)
Black	6,495 (10.7%)	3,485 (8.8%)	6,270 (11.7%)
Other	11,770 (19.3%)	5,710 (14.4%)	8,860 (16.5%)
Missing	2,025 (3.3%)	1,190 (3.0%)	2,635 (4.9%)
Primary payer, N (%)			
Medicare	25,155 (41.3%)	20,130 (50.8%)	23,185 (43.2%)
Private Insurance	23,880 (39.2%)	14,435 (36.5%)	19,370 (36.1%)
Medicaid	4,835 (7.9%)	3,000 (7.6%)	5,710 (10.6%)
Other	6,765 (11.1%)	1,950 (4.9%)	5,235 (9.8%)
Missing	220 (0.4%)	75 (0.2%)	170 (0.3%)
Zip Code median income quartile, N (%)			
Q1	14,495 (23.8%)	9,250 (23.4%)	14,225 (26.5%)
Q2	15,320 (25.2%)	9,950 (25.1%)	14,440 (26.9%)
Q3	15,180 (24.93%)	10,245 (25.9%)	13,200 (24.6%)
Q4	14,895 (24.5%)	9,605 (24.3%)	10,880 (20.3%)
Missing	965 (1.6%)	540 (1.4%)	925 (1.7%)
ECI, N (%)			
0	18,790 (30.9%)	7,685 (19.4%)	12,610 (23.5%)
1	17,295 (28.4%)	11,320 (28.6%)	14,830 (27.6%)
2	13,645 (22.4%)	10,745 (27.1%)	13,560 (25.3%)
3 or greater	11,125 (18.3%)	9,840 (24.9%)	12,670 (23.6%)

Percents may not add to 100 due to rounding

LARN = laparoscopic radical nephrectomy; RARN = robotic-assisted radical nephrectomy; ORN = open radical nephrectomy; Q1, Q2, Q3, and Q4 = income quartile; ECI = Elixhauser Comorbidity Index

Patient characteristics varied across surgical approaches. Compared to LARN and ORN, RARN patients were older, predominantly male, white, and had more comorbidities (Table 1).

The distributions of hospital characteristics, including hospital type, region, and bed size, showed no major differences among patients by surgical techniques (Table 2). However, LARN was more frequently performed at high-volume hospitals (35.5%) compared to RARN (26.5%) and ORN (28.8%).

**Table 2:** Hospital characteristics of patients by surgical technique

	LARN	RARN	ORN
Patients, N (%)	60,855 (39.5)	39,590 (25.7)	53,670 (34.8)
Hospital type, N (%)			
Urban (teaching)	51,730 (85.0%)	33,465 (84.5%)	44,230 (82.4%)
Urban (nonteaching)	7,725 (12.7%)	5,140 (13.0%)	7,595 (14.2%)
Rural	1,400 (2.3%)	985 (2.5%)	1,845 (3.4%)
Hospital region (%)			
Northeast	12,105 (19.9%)	7,035 (17.8%)	8,495 (15.8%)
Midwest	10,615 (17.4%)	11,180 (28.2%)	12,485 (23.3%)
South	24,325 (40.0%)	13,560 (34.3%)	22,735 (42.4%)
West	13,810 (22.7%)	7,815 (19.7%)	9,955 (18.5%)
Hospital bed size (%)			
Small	8,760 (14.4%)	5,450 (13.8%)	6,505 (12.1%)
Medium	14,110 (23.2%)	9,925 (25.1%)	12,925 (24.1%)
Large	37,985 (62.4%)	24,215 (61.2%)	34,240 (63.8%)
Hospital surgical volume (%)			
Small	8,760 (14.4%)	5,450 (13.8%)	6,505 (12.1%)
Intermediate	29,390 (48.3%)	22,945 (58.0%)	27,760 (51.7%)
High	21,595 (35.5%)	10,480 (26.5%)	15,435 (28.8%)

Percents may not add to 100 due to rounding

LARN = laparoscopic radical nephrectomy; RARN = robotic-assisted radical nephrectomy; ORN = open radical nephrectomy

Table 3 presents intraoperative and inpatient postoperative patient outcomes. Blood transfusions were more common in ORN (11.8%) than in RARN (3.8%) and LARN (2.9%). Postoperative complications were also higher in ORN (15.9%) compared to RARN (8.0%) and LARN (7.2%). Hospital mortality rates were highest for ORN (3.0%), whereas RARN and LARN had significantly lower rates (0.2% and 0.3%, respectively). Median length of stay (LOS) was longest for ORN.

**Table 3:** Patient intraoperative and inpatient postoperative outcomes during hospitalization by surgical technique

	LARN	RARN	ORN
Patients, N (%)	60,855 (39.5)	39,590 (25.7)	53,670 (34.8)
Blood transfusions, N (%)	1,780 (2.9%)	1,490 (3.8%)	6,350 (11.8%)
Any complications, N (%)	4,360 (7.2%)	3,160 (8.0%)	8,530 (15.9%)
Cardiac	265 (0.4%)	200 (0.5%)	485 (0.9%)
Genitourinary	900 (1.5%)	655 (1.7%)	1,775 (3.3%)
Respiratory	605 (1.0%)	610 (1.5%)	1,920 (3.6%)
Vascular	65 (0.1%)	65 (0.2%)	285 (0.5%)
Wound or Infection	690 (1.1%)	550 (1.4%)	2,125 (4.0%)
Bleeding	90 (0.1%)	60 (0.2%)	235 (0.4%)
Misc. Medical	35 (0.1%)	30 (0.1%)	40 (0.1%)
Misc. Surgical	2,370 (3.9%)	1,580 (4.0%)	4,130 (7.7%)
Number of complications, N (%)			
0	56,495 (92.8%)	36,430 (92.0%)	45,140 (84.1%)
1	3,680 (6.0%)	2,565 (6.5%)	6,205 (11.6%)
2+	680 (1.1%)	595 (1.5%)	2,325 (4.3%)
Discharge status, N (%)			
Routine	54,055 (88.8%)	33,930 (85.7%)	41,220 (76.8%)
Non-Routine	6,575 (10.8%)	5,540 (14.0%)	10,820 (20.2%)
Missing	225 (0.4%)	120 (0.3%)	1,630 (3.0%)
Death, N (%)	185 (0.3%)	90 (0.2%)	1,600 (3.0%)
LOS (days), Median [Q1, Q3]	2 [2, 4]	2 [2, 4]	4 [3, 7]
Total costs ($), Median [Q1, Q3]	13,950 [10,628−18,924]	16,771 [12,794−22,794]	17,821 [12,703−28,516]

Percents may not add to 100 due to rounding

LARN = laparoscopic radical nephrectomy; RARN = robotic-assisted radical nephrectomy; ORN = open radical nephrectomy; LOS = length of stay; Q1, Q3 = first and third interquartiles

**Fig. 1:**
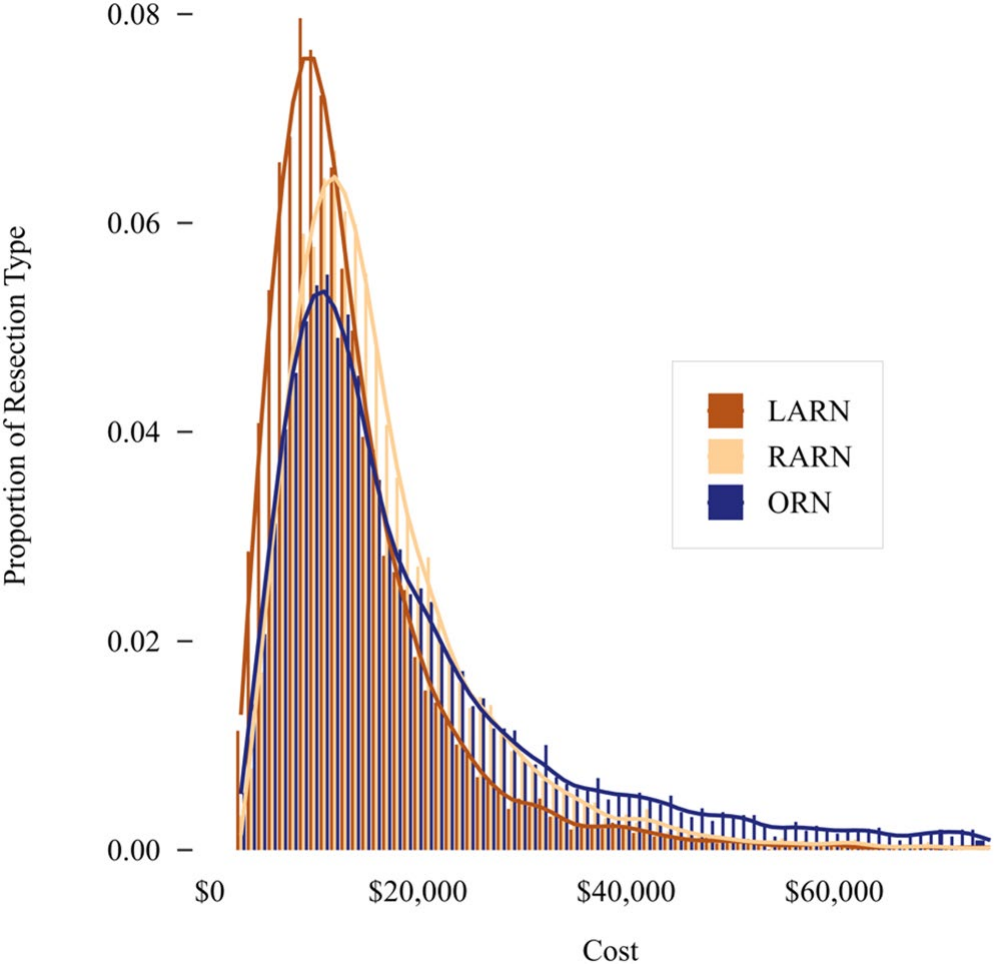
Distribution of total hospital costs by surgical technique. LARN = laparoscopic radical nephrectomy; RARN = robotic-assisted radical nephrectomy; ORN = open radical nephrectomy

Hospital costs were right-skewed and varied across procedures (Fig. 1), with median costs of $17,821 [Q1-Q3: 12,703–28,516] for ORN, $16,771 [Q1-Q3: 12,794–22,794] for RARN, and $13,950 [Q1-Q3: 10,628–18,924] for LARN.

Multivariable logistic regression analysis showed that LARN and RARN patients were significantly less likely to require blood transfusions or experience inpatient postoperative complications compared to those undergoing ORN (Table 4; Supplemental Tables 2–10). Poisson regression indicated that LARN (IRR 0.82; 95% CI 0.71–0.95; *p* = 0.007) and RARN (IRR 0.82; 95% CI 0.70–0.96; *p* = 0.014) were each associated with 18% lower complication counts compared to ORN (Table 4; Supplemental Table 11). Patients undergoing minimally invasive procedures also had about 50% lower odds of non-routine discharge (Table 4; Supplemental Table 9). Mortality odds were significantly lower for LARN (OR 0.10; 95% CI 0.07–0.15; *p* < 0.0001) and RARN (OR 0.08; 95% CI 0.05–0.14; *p* < 0.0001) compared to ORN (Table 4; Supplemental Table 10).

Negative binomial regression showed about 20% lower LOS counts for LARN (IRR 0.80; 95% CI 0.76–0.84; *p* < 0.0001) and RARN (IRR 0.77; 95% CI 0.73–0.80; *p* < 0.0001) relative to ORN (Table 4; Supplemental Table 12). Linear regression indicated that LARN and RARN were respectively 27% (e^β^ 0.73; 95% CI 0.72–0.74; *p* < 0.0001) and 15% (e^β^ 0.85; 95% CI 0.84–0.87; *p* < 0.0001) less costly than ORN, on average (Table 4; Supplemental Table 13).

**Table 4:** Regression analyses of LARN and RARN vs. ORN

Multivariable logistic regression analysis of LARN and RARN vs. ORN intraoperative and postoperative outcome			
	Adjusted OR	95% CI	*p*-value
Blood transfusions			
LARN	0.23	0.20–0.27	< 0.0001
RARN	0.28	0.24–0.32	< 0.0001
Any complications			
LARN	0.43	0.40–0.47	< 0.0001
RARN	0.43	0.39–0.48	< 0.0001
Cardiac complication			
LARN	0.49	0.35–0.69	< 0.0001
RARN	0.50	0.34–0.74	0.0005
Genitourinary complication			
LARN	0.48	0.40–0.57	< 0.0001
RARN	0.46	0.38–0.57	< 0.0001
Respiratory complicatio			
LARN	0.29	0.23–0.36	< 0.0001
RARN	0.41	0.33–0.50	< 0.0001
Wound/Infection complication			
LARN	0.30	0.24–0.36	< 0.0001
RARN	0.33	0.27–0.41	< 0.0001
Misc. Surgical complication			
LARN	0.52	0.46–0.59	< 0.0001
RARN	0.46	0.40–0.53	< 0.0001
Non-routine discharge			
LARN	0.49	0.45–0.53	< 0.0001
RARN	0.52	0.48–0.57	< 0.0001
Death			
LARN	0.10	0.07–0.15	< 0.0001
RARN	0.08	0.05–0.14	< 0.0001

Poisson regression analysis of LARN and RARN vs. ORN for complication counts			
	IRR	95% CI	*p*-value
LARN	0.82	0.71–0.95	0.007
RARN	0.82	0.70–0.96	0.014

Negative binomial regression analysis of LAPN and RAPN vs. OPN for LOS			
	IRR	95% CI	*p*-value
LARN	0.80	0.76–0.84	< 0.0001
RARN	0.77	0.73–0.80	< 0.0001

Linear regression analysis of LAPN and RAPN vs. OPN for hospital costs			
	e^β^	95% CI	*p*-value
LARN	0.73	0.72–0.74	< 0.0001
RARN	0.85	0.84–0.87	< 0.0001

Models adjusted for patient characteristics (sex, age, race, Zip code median income, primary payer, and comorbidity index) and hospital characteristics (type, region, and volume)

LARN = laparoscopic radical nephrectomy; RARN = robotic-assisted radical nephrectomy, ORN = open radical nephrectomy, LOS = length of stay;, OR = odds ratio; CI = confidence interval

In a secondary analysis, RARN and LARN were compared across all outcomes using the same regression models. There were no statistically significant differences across most of the outcomes (Supplemental Tables 14 and 15). Patients undergoing RARN were significantly more likely to experience inpatient respiratory complications (RARN OR 1.37; 95% CI 1.05–1.78; *p* = 0.02). RARN was also 15% more costly (e^β^ 1.15; 95% CI 1.14–1.17; *p* < 0.0001) on average but showed 5% lower LOS counts (IRR 0.95; 95% CI 0.92–0.99; *p* < 0.008) as compared to LARN.

## Discussion

Our study provides a contemporary analysis of the utilization, inpatient perioperative outcomes, and economic impact of LARN and RARN compared to ORN in patients with renal cancer. Minimally invasive approaches were the predominant surgical techniques during the study period, with RARN utilization increasing (21.9% to 29.9%) and LARN correspondingly declining (45.6% to 35.6%). Both RARN and LARN were associated with more favorable inpatient perioperative outcomes relative to ORN, including lower rates of complications, reduced need for blood transfusions, and shorter hospital stays – each of which might partly explain the lower costs associated with RARN and LARN. However, despite comparable inpatient perioperative outcomes, RARN remained significantly more costly than LARN.

The rising preference for RARN alongside the decline in LARN may reflect broader trends favoring robotic platforms in urologic oncology. This shift may be driven by multiple factors, including surgeon preference, institutional investment in robotic systems, and evolving training paradigms that emphasize robotic proficiency. Notably, LARN was more frequently performed at high-volume hospitals in our cohort, which may indicate that surgeons in these centers had developed laparoscopic expertise earlier and were able to proctor others in adopting minimally invasive approaches. The institutional learning environment and structured proctoring programs typical of high-volume hospitals may also have accelerated the transition to robotic platforms, facilitating broader dissemination of surgical innovation and training in minimally invasive techniques [18]. Interestingly, RARN was more frequently performed in older and more comorbid patients, suggesting a balanced clinical approach when choosing robotic techniques for higher-risk populations.

Our findings align with previous research showing better perioperative outcomes for minimally invasive techniques compared to ORN, particularly in reducing postoperative morbidity, hospital LOS, and transfusion requirements [3–6]. Prior studies have also documented a steady rise in RARN adoption with a concurrent decline in LARN, attributing this to robotic articulation and high-definition visualization that enhance surgical dexterity [6, 7, 9]. Nonetheless, our findings reaffirm prior evidence indicating that, while RARN may be technologically advantageous, it does not confer significantly better perioperative outcomes than LARN and remains the more expensive option [6, 9, 19, 20].

Several economic evaluations have attributed the increased costs of robotic-assisted procedures, attributing them to longer operative times, higher instrument expenses, and the specialized training requirements [3, 8]. Despite these constraints, the use of robotic surgery continues to increase in popularity. Urology was an early adopter of robotic technology and recent work predicted that by 2025 robotic approaches would increase in favor over laparoscopic and open approaches among other surgical specialties [21]. An increase that has persisted in the face of financial concerns that have been raised since the early adoption of robotics for clinical practice [22]. This sustained momentum likely reflects not only technical and ergonomic benefits but also cultural and systemic drivers—such as surgeon familiarity, institutional investment, and increasing exposure in residency programs—that collectively reinforce robotic surgery as the perceived standard of care among newer generations of surgeons [23].

The high acquisition and maintenance costs of robotic systems raise questions about the financial sustainability of RARN across healthcare settings. For budget-constrained institutions, LARN may offer comparable outcomes with lower costs. However, in complex cases where RARN’s precision may improve safety and reduce complications, its higher cost may be justified.

This study has several limitations. The NIS administrative dataset does not include granular clinical and operative details, such as tumor stage, size, histology, radiological findings, and intraoperative parameters, which may have influenced both surgical selection and patient outcomes. Because ORN is typically preferred for larger or more complex tumors, cases involving inferior vena cava thrombus, or patients with substantial comorbidities burdens, observed differences in outcomes and costs between minimally invasive and ORN may in part reflect underlying disease severity rather than the surgical technique itself [24, 25]. Unfortunately, these clinical nuances could not be captured within the NIS database. The NIS also does not include a standardized grading system for perioperative complications, such as the Clavien–Dindo classification. Consequently, complications could not be stratified by severity, limiting the depth of comparative safety assessment. In addition, the dataset does not identify cases that were initiated as robotic-assisted or laparoscopic nephrectomies but were converted intraoperatively to open surgery. This limitation could have introduced selection bias and potentially overstated the comparative advantages of minimally invasive approaches, since converted cases—often representing more complex surgeries—would be misclassified as open procedures. Potential inaccuracies inherent to large administrative databases, such as miscoded or missing data, could have introduced bias, affecting the reliability of our findings. Furthermore, the dataset does not provide surgeon-level characteristics, even though operator expertise is a key determinant of perioperative outcomes.

The absence of patient-reported outcomes may have limited the ability to comprehensively assess oncological efficacy and quality-of-life implications in our comparisons of procedures. Our cost estimates for RARN were likely conservative, as the NIS does not capture capital expenditures for robotic systems or their maintenance, which are major cost drivers. The introduction of newer-generation robotic platforms may further amplify these costs, underscoring the need for ongoing economic evaluations as technology continues to evolve. Moreover, the 2016–2019 study window may not fully reflect contemporary clinical and economic practice patterns, particularly given the likely impact of the COVID-19 pandemic in accelerating minimally invasive adoption. Validation with more recent, post-pandemic data is warranted. Lastly, while this analysis focused on hospital-level costs, a formal cost-effectiveness evaluation would be necessary to fully understand the economic implications of RARN versus LARN and determine whether the increased costs of RARN were justified by potential long-term benefits.

Further investigations using datasets with richer clinical detail, including tumor characteristics and long-term patient outcomes, are necessary to enhance the robustness and generalizability of this study’s results. Future studies should also prioritize the evaluation of patient-centered outcomes, such as health-related quality of life and survival over extended periods, to fully assess the value provided by robotic-assisted techniques. Cost-effectiveness analyses specific to distinct demographic groups, such as older patients or those with significant comorbidities, would be valuable in identifying patient populations that might derive the most benefit from RARN. These focused analyses could support evidence-based resource allocation and informed clinical decision-making based on patient-specific characteristics and healthcare system constraints.

Utilization of laparoscopic and robotic approaches varies by specialty. General surgery and gynecology were early adopters of laparoscopy and continue to rely on it [26]. Urology, after initially adopting laparoscopy, has more extensively transitioned to robotic surgery, potentially influencing adoption in other fields [27]. Regardless, evaluating the cost-effectiveness of robotic versus laparoscopic approaches remains essential to guide clinical and policy decisions.

## Conclusions

In conclusion, our study highlights the continued shift toward RARN in radical nephrectomy despite its higher costs and comparable inpatient perioperative outcomes to LARN. While both minimally invasive techniques demonstrated advantages over ORN, the economic burden associated with RARN might be an important consideration for policy and clinical decisions. Future research should focus on long-term outcomes, cost-effectiveness, and institutional factors driving the preference for robotic surgery. More comprehensive assessments of oncologic outcomes and patient-centered measures are warranted to guide evidence-based decision-making in surgical approach selection for renal cancer patients.

## Supplementary Information

Below is the link to the electronic supplementary material.

Supplementary Material 1

## Data Availability

The data underlying this study are not publicly available due to restrictions imposed by the Data Use Agreement governing the Healthcare Cost and Utilization Project (HCUP) Nationwide Databases, executed between V. M. and the Agency for Healthcare Research and Quality (AHRQ). Access to these data is limited to qualified researchers through AHRQ’s established procedures. For details on how to obtain HCUP data, please visit the AHRQ HCUP website at [www.hcup-us.ahrq.gov](http:/www.hcup-us.ahrq.gov).

## Abbreviations

LARN: Laparoscopic radical nephrectomy
RARN: Robotic-assisted radical nephrectomy
ORN: Open radical nephrectomy
LOS: Length of stay
RCC: Renal cell carcinoma
NIS: National Inpatient Sample
HCUP: Healthcare Cost and Utilization Project
AHRQ: Agency for Healthcare Research and Quality
ICD-10-CM: International Classification of Diseases-10 Clinical Modification
PCS: Procedure Coding System
ECI: Elixhauser Comorbidity Index
