# Supplementary material for: Comparative analysis of Robotic-Assisted, Laparoscopic, and open radical nephrectomy: Utilization, Costs, and clinical outcomes
Source: J Robot Surg. 2025 Nov 24;20(1):41. doi: 10.1007/s11701-025-02995-x (PMC12641040; doi:10.1007/s11701-025-02995-x)

**Supplementary Material**

**Submitted to the Journal of Robotic Surgery**

**Comparative Analysis of Robotic-Assisted, Laparoscopic, and Open Radical Nephrectomy: Utilization, Costs, and Clinical Outcomes**

Daniel Y. Huang, PharmD,^a^ Costas D. Lallas, MD, FACS,^b^ Raegan M. Davis, MA,^a^ Scott W. Keith, PhD,^c,d^ Patrick J. Moeller, MPH,^a^ Inkyu K. Kim, PhD,^a^ Anushka Ghosh, BS,^b^ Francisco Aguirre, BS,^b^ Rasheed A. M. Thompson, BS,^b^ Emmanuel F. Drabo, MPhil, PhD,^e^ Vittorio Maio, PharmD, MS, MSPH^a,f^

**Corresponding Author**:

Vittorio Maio, PharmD, MS, MSPH

College of Population Health

Thomas Jefferson University

901 Walnut St., 10^th^ Floor, Philadelphia, Pennsylvania, 19107, USA

Phone: (215) 955-1821

Fax: (215) 503-7598

Email: [vittorio.maio@jefferson.edu](mailto:vittorio.maio@jefferson.edu)

| **Supplemental Table 1. ICD-10-CM and PCS procedure codes for ascertaining complications** | |
| --- | --- |
| *Complication category* | *ICD-10-CM and PCS procedure codes* |
| Blood transfusion | 302 |
| Cardiac complications | I970, I971, I977, I978 |
| Genitourinary complications | T83, T8140, T8141, T8142, T8143, T8149, T83, N99 |
| Respiratory complications | J95 |
| Vascular complications | T817, T882, I973, I974, I976 |
| Wound or infection complications | T813, T814, I975, I976, L761, L762, E361, K917, D78 |
| Bleeding complications | L760, L763, E360, K916 |
| Miscellaneous medical complications | T883, T884, T885, T886, T887, R5082 |
| Miscellaneous surgical complications | T811, T815, T816, T818, T819, T888, T889, D78, L768, E368, M96, K910, K911, K912, K913, K915, K918, T80, E89 |

| **Supplemental Table 2. Multivariable logistic regression analysis of LARN and RARN vs ORN for blood transfusions** | | | |
| --- | --- | --- | --- |
|  | Adjusted OR | 95% CI | p-value |
| ORN (referent) |  |  |  |
| LARN | 0.23 | 0.20-0.27 | < 0.0001 |
| RARN | 0.28 | 0.24-0.32 | < 0.0001 |
| Age:  ≤50 (referent) |  |  |  |
| >50 | 1.11 | 0.99-1.26 | 0.10 |
| Sex:  Male (referent) |  |  |  |
| Female | 1.05 | 0.95-1.16 | 0.35 |
| Race:  White (referent) |  |  |  |
| Non-White | 1.68 | 1.51-1.87 | < 0.0001 |
| Primary payer:  Private insurance (referent) |  |  |  |
| Non-Private payer | 1.38 | 1.23-1.54 | < 0.0001 |
| Zip code median income:  Q4 (referent) |  |  |  |
| Q1-Q3 | 0.81 | 0.71-0.91 | 0.001 |
| ECI (per count) | 1.25 | 1.21-1.30 | < 0.0001 |
| Hospital type:  Urban teaching (referent) |  |  |  |
| Other hospital type | 0.85 | 0.74-0.99 | 0.03 |
| Hospital region:  South/Midwest/West (referent) |  |  |  |
| North | 0.87 | 0.77-0.99 | 0.06 |
| Hospital volume:  High (referent) |  |  |  |
| Low/intermediate | 1.33 | 1.13-1.55 | 0.0002 |

OR=odds ratio; LARN=laparoscopic radical nephrectomy; RARN=robotic-assisted radical nephrectomy; ORN=open radical nephrectomy; Non-White=Black, Other; Non-Private payer=Medicare, Medicaid, Other; Q1, Q2, Q3, and Q4=income quartile; ECI=Elixhauser Comorbidity Index; Other hospital type=Urban (nonteaching), Rural

| **Supplemental Table 3. Multivariable logistic regression analysis of LARN and RARN vs ORN for any complications** | | | |
| --- | --- | --- | --- |
|  | Adjusted OR | 95% CI | p-value |
| ORN (referent) |  |  |  |
| LARN | 0.43 | 0.40-0.47 | < 0.0001 |
| RARN | 0.43 | 0.39-0.48 | < 0.0001 |
| Age:  ≤50 (referent) |  |  |  |
| >50 | 1.18 | 1.06-1.30 | 0.002 |
| Sex:  Male (referent) |  |  |  |
| Female | 0.90 | 0.83-0.97 | 0.006 |
| Race:  White (referent) |  |  |  |
| Non-White | 1.05 | 0.97-1.15 | 0.23 |
| Primary payer:  Private insurance (referent) |  |  |  |
| Non-Private payer | 1.21 | 1.11-1.31 | < 0.0001 |
| Zip code median income:  Q4 (referent) |  |  |  |
| Q1-Q3 | 1.00 | 0.91-1.10 | 0.99 |
| ECI (per count) | 1.26 | 1.22-1.30 | < 0.0001 |
| Hospital type:  Urban teaching (referent) |  |  |  |
| Other hospital type | 1.08 | 0.98-1.20 | 0.14 |
| Hospital region:  South/Midwest/West (referent) |  |  |  |
| North | 1.14 | 1.05-1.24 | 0.002 |
| Hospital volume:  High (referent) |  |  |  |
| Low/intermediate | 1.21 | 1.10-1.33 | 0.0001 |

OR=odds ratio; LARN=laparoscopic radical nephrectomy; RARN=robotic-assisted radical nephrectomy; ORN=open radical nephrectomy; Non-White=Black, Other; Non-Private payer=Medicare, Medicaid, Other; Q1, Q2, Q3, and Q4=income quartile; ECI=Elixhauser Comorbidity Index; Other hospital type=Urban (nonteaching), Rural

| **Supplemental Table 4. Multivariable logistic regression analysis of LARN and RARN vs ORN for cardiac complications** | | | |
| --- | --- | --- | --- |
|  | Adjusted OR | 95% CI | p-value |
| ORN (referent) |  |  |  |
| LARN | 0.49 | 0.35-0.69 | < 0.0001 |
| RARN | 0.50 | 0.34-0.74 | 0.0005 |
| Age:  ≤50 (referent) |  |  |  |
| >50 | 1.71 | 1.11-2.63 | 0.014 |
| Sex:  Male (referent) |  |  |  |
| Female | 0.52 | 0.37-0.72 | 0.0001 |
| Race:  White (referent) |  |  |  |
| Non-White | 1.08 | 0.78-1.48 | 0.66 |
| Primary payer:  Private insurance (referent) |  |  |  |
| Non-Private payer | 1.34 | 0.95-1.90 | 0.09 |
| Zip code median income:  Q4 (referent) |  |  |  |
| Q1-Q3 | 1.05 | 0.73-1.49 | 0.80 |
| ECI (per count) | 1.11 | 0.98-1.25 | 0.10 |
| Hospital type:  Urban teaching (referent) |  |  |  |
| Other hospital type | 0.76 | 0.49-1.19 | 0.23 |
| Hospital region:  South/Midwest/West (referent) |  |  |  |
| North | 1.14 | 0.84-1.54 | 0.42 |
| Hospital volume:  High (referent) |  |  |  |
| Low/intermediate | 0.89 | 0.66-1.22 | 0.48 |

OR=odds ratio; LARN=laparoscopic radical nephrectomy; RARN=robotic-assisted radical nephrectomy; ORN=open radical nephrectomy; Non-White=Black, Other; Non-Private payer=Medicare, Medicaid, Other; Q1, Q2, Q3, and Q4=income quartile; ECI=Elixhauser Comorbidity Index; Other hospital type=Urban (nonteaching), Rural

| **Supplemental Table 5. Multivariable logistic regression analysis of LARN and RARN vs ORN for genitourinary complications** | | | |
| --- | --- | --- | --- |
|  | Adjusted OR | 95% CI | p-value |
| ORN (referent) |  |  |  |
| LARN | 0.48 | 0.40-0.57 | < 0.0001 |
| RARN | 0.46 | 0.38-0.57 | < 0.0001 |
| Age:  ≤50 (referent) |  |  |  |
| >50 | 1.00 | 0.82-1.24 | 0.97 |
| Sex:  Male (referent) |  |  |  |
| Female | 0.89 | 0.76-1.05 | 0.16 |
| Race:  White (referent) |  |  |  |
| Non-White | 1.11 | 0.94-1.32 | 0.23 |
| Primary payer:  Private insurance (referent) |  |  |  |
| Non-Private payer | 1.25 | 1.04-1.49 | 0.02 |
| Zip code median income:  Q4 (referent) |  |  |  |
| Q1-Q3 | 0.93 | 0.76-1.12 | 0.43 |
| ECI (per count) | 1.24 | 1.17-1.31 | < 0.0001 |
| Hospital type:  Urban teaching (referent) |  |  |  |
| Other hospital type | 1.13 | 0.92-1.38 | 0.26 |
| Hospital region:  South/Midwest/West (referent) |  |  |  |
| North | 1.18 | 1.00-1.40 | 0.05 |
| Hospital volume:  High (referent) |  |  |  |
| Low/intermediate | 1.33 | 1.09-1.63 | 0.005 |

OR=odds ratio; LARN=laparoscopic radical nephrectomy; RARN=robotic-assisted radical nephrectomy; ORN=open radical nephrectomy; Non-White=Black, Other; Non-Private payer=Medicare, Medicaid, Other; Q1, Q2, Q3, and Q4=income quartile; ECI=Elixhauser Comorbidity Index; Other hospital type=Urban (nonteaching), Rural

| **Supplemental Table 6. Multivariable logistic regression analysis of LARN and RARN vs ORN for respiratory complications** | | | |
| --- | --- | --- | --- |
|  | Adjusted OR | 95% CI | p-value |
| ORN (referent) |  |  |  |
| LARN | 0.29 | 0.23-0.36 | < 0.0001 |
| RARN | 0.41 | 0.33-0.50 | < 0.0001 |
| Age:  ≤50 (referent) |  |  |  |
| >50 | 1.16 | 0.94-1.44 | 0.17 |
| Sex:  Male (referent) |  |  |  |
| Female | 0.98 | 0.84-1.16 | 0.83 |
| Race:  White (referent) |  |  |  |
| Non-White | 1.20 | 1.00-1.44 | 0.05 |
| Primary payer:  Private insurance (referent) |  |  |  |
| Non-Private payer | 1.33 | 1.10-1.61 | 0.003 |
| Zip code median income:  Q4 (referent) |  |  |  |
| Q1-Q3 | 1.03 | 0.84-1.27 | 0.77 |
| ECI (per count) | 1.35 | 1.27-1.43 | < 0.0001 |
| Hospital type:  Urban teaching (referent) |  |  |  |
| Other hospital type | 1.11 | 0.90-1.38 | 0.32 |
| Hospital region:  South/Midwest/West (referent) |  |  |  |
| North | 1.10 | 0.92-1.31 | 0.28 |
| Hospital volume:  High (referent) |  |  |  |
| Low/intermediate | 1.38 | 1.12-1.70 | 0.003 |

OR=odds ratio; LARN=laparoscopic radical nephrectomy; RARN=robotic-assisted radical nephrectomy; ORN=open radical nephrectomy; Non-White=Black, Other; Non-Private payer=Medicare, Medicaid, Other; Q1, Q2, Q3, and Q4=income quartile; ECI=Elixhauser Comorbidity Index; Other hospital type=Urban (nonteaching), Rural

| **Supplemental Table 7. Multivariable logistic regression analysis of LARN and RARN vs ORN for wound/infection complications** | | | |
| --- | --- | --- | --- |
|  | Adjusted OR | 95% CI | p-value |
| ORN (referent) |  |  |  |
| LARN | 0.30 | 0.24-0.36 | < 0.0001 |
| RARN | 0.33 | 0.27-0.41 | < 0.0001 |
| Age:  ≤50 (referent) |  |  |  |
| >50 | 1.35 | 1.09-1.67 | 0.006 |
| Sex:  Male (referent) |  |  |  |
| Female | 0.92 | 0.79-1.08 | 0.30 |
| Race:  White (referent) |  |  |  |
| Non-White | 1.34 | 1.13-1.58 | 0.0007 |
| Primary payer:  Private insurance (referent) |  |  |  |
| Non-Private payer | 1.12 | 0.94-1.33 | 0.19 |
| Zip code median income:  Q4 (referent) |  |  |  |
| Q1-Q3 | 1.03 | 0.85-1.26 | 0.75 |
| ECI (per count) | 1.19 | 1.12-1.26 | < 0.0001 |
| Hospital type:  Urban teaching (referent) |  |  |  |
| Other hospital type | 1.02 | 0.83-1.25 | 0.86 |
| Hospital region:  South/Midwest/West (referent) |  |  |  |
| North | 1.01 | 0.86-1.19 | 0.92 |
| Hospital volume:  High (referent) |  |  |  |
| Low/intermediate | 1.10 | 0.92-1.33 | 0.29 |

OR=odds ratio; LARN=laparoscopic radical nephrectomy; RARN=robotic-assisted radical nephrectomy; ORN=open radical nephrectomy; Non-White=Black, Other; Non-Private payer=Medicare, Medicaid, Other; Q1, Q2, Q3, and Q4=income quartile; ECI=Elixhauser Comorbidity Index; Other hospital type=Urban (nonteaching), Rural

| **Supplemental Table 8. Multivariable logistic regression analysis of LARN and RARN vs ORN for misc. surgical complications** | | | |
| --- | --- | --- | --- |
|  | Adjusted OR | 95% CI | p-value |
| ORN (referent) |  |  |  |
| LARN | 0.52 | 0.46-0.59 | < 0.0001 |
| RARN | 0.46 | 0.40-0.53 | < 0.0001 |
| Age:  ≤50 (referent) |  |  |  |
| >50 | 1.31 | 1.13-1.51 | 0.0003 |
| Sex:  Male (referent) |  |  |  |
| Female | 0.87 | 0.79-0.97 | 0.01 |
| Race:  White (referent) |  |  |  |
| Non-White | 0.90 | 0.80-1.02 | 0.10 |
| Primary payer:  Private insurance (referent) |  |  |  |
| Non-Private payer | 1.21 | 1.08-1.36 | 0.001 |
| Zip code median income:  Q4 (referent) |  |  |  |
| Q1-Q3 | 1.01 | 0.89-1.15 | 0.87 |
| ECI (per count) | 1.22 | 1.17-1.27 | < 0.0001 |
| Hospital type:  Urban teaching (referent) |  |  |  |
| Other hospital type | 1.05 | 0.92-1.21 | 0.48 |
| Hospital region:  South/Midwest/West (referent) |  |  |  |
| North | 1.22 | 1.09-1.36 | 0.0008 |
| Hospital volume:  High (referent) |  |  |  |
| Low/intermediate | 1.25 | 1.10-1.43 | 0.001 |

OR=odds ratio; LARN=laparoscopic radical nephrectomy; RARN=robotic-assisted radical nephrectomy; ORN=open radical nephrectomy; Non-White=Black, Other; Non-Private payer=Medicare, Medicaid, Other; Q1, Q2, Q3, and Q4=income quartile; ECI=Elixhauser Comorbidity Index; Other hospital type=Urban (nonteaching), Rural

| **Supplemental Table 9. Multivariable logistic regression analysis of LARN and RARN vs ORN for non-routine discharge** | | | |
| --- | --- | --- | --- |
|  | Adjusted OR | 95% CI | p-value |
| ORN (referent) |  |  |  |
| LARN | 0.49 | 0.45-0.53 | < 0.0001 |
| RARN | 0.52 | 0.48-0.57 | < 0.0001 |
| Age:  ≤50 (referent) |  |  |  |
| >50 | 2.48 | 2.23-2.77 | < 0.0001 |
| Sex:  Male (referent) |  |  |  |
| Female | 1.20 | 1.17-1.28 | < 0.0001 |
| Race:  White (referent) |  |  |  |
| Non-White | 1.01 | 0.93-1.09 | 0.89 |
| Primary payer:  Private insurance (referent) |  |  |  |
| Non-Private payer | 2.80 | 2.56-3.05 | < 0.0001 |
| Zip code median income:  Q4 (referent) |  |  |  |
| Q1-Q3 | 0.97 | 0.89-1.05 | 0.43 |
| ECI (per count) | 1.43 | 1.39-1.47 | < 0.0001 |
| Hospital type:  Urban teaching (referent) |  |  |  |
| Other hospital type | 1.00 | 0.91-1.10 | 0.95 |
| Hospital region:  South/Midwest/West (referent) |  |  |  |
| North | 1.29 | 1.19-1.40 | < 0.0001 |
| Hospital volume:  High (referent) |  |  |  |
| Low/intermediate | 1.05 | 0.95-1.16 | 0.35 |

OR=odds ratio; LARN=laparoscopic radical nephrectomy; RARN=robotic-assisted radical nephrectomy; ORN=open radical nephrectomy; Non-White=Black, Other; Non-Private payer=Medicare, Medicaid, Other; Q1, Q2, Q3, and Q4=income quartile; ECI=Elixhauser Comorbidity Index; Other hospital type=Urban (nonteaching), Rural

| **Supplemental Table 10. Multivariable logistic regression analysis of LARN and RARN vs ORN for death** | | | |
| --- | --- | --- | --- |
|  | Adjusted OR | 95% CI | p-value |
| ORN (referent) |  |  |  |
| LARN | 0.10 | 0.07-0.15 | < 0.0001 |
| RARN | 0.08 | 0.05-0.14 | < 0.0001 |
| Age:  ≤50 (referent) |  |  |  |
| >50 | 0.81 | 0.63-1.02 | 0.08 |
| Sex:  Male (referent) |  |  |  |
| Female | 0.61 | 0.48-0.77 | < 0.0001 |
| Race:  White (referent) |  |  |  |
| Non-White | 1.60 | 1.23-2.00 | < 0.0001 |
| Primary payer:  Private insurance (referent) |  |  |  |
| Non-Private payer | 1.36 | 1.03-1.80 | 0.03 |
| Zip code median income:  Q4 (referent) |  |  |  |
| Q1-Q3 | 1.44 | 1.06-1.96 | 0.02 |
| ECI (per count) | 0.80 | 0.70-0.91 | 0.0009 |
| Hospital type:  Urban teaching (referent) |  |  |  |
| Other hospital type | 0.80 | 0.59-1.10 | 0.17 |
| Hospital region:  South/Midwest/West (referent) |  |  |  |
| North | 0.82 | 0.63-1.08 | 0.16 |
| Hospital volume:  High (referent) |  |  |  |
| Low/intermediate | 2.25 | 1.63-3.10 | < 0.0001 |

OR=odds ratio; LARN=laparoscopic radical nephrectomy; RARN=robotic-assisted radical nephrectomy; ORN=open radical nephrectomy; Non-White=Black, Other; Non-Private payer=Medicare, Medicaid, Other; Q1, Q2, Q3, and Q4=income quartile; ECI=Elixhauser Comorbidity Index; Other hospital type=Urban (nonteaching), Rural

| **Supplemental Table 11. Multivariable Poisson regression analysis of LARN and RARN vs ORN for complication counts** | | | |
| --- | --- | --- | --- |
|  | Adjusted IRR | 95% CI | p-value |
| ORN (referent) |  |  |  |
| LARN | 0.82 | 0.71-0.95 | 0.007 |
| RARN | 0.82 | 0.70-0.96 | 0.014 |
| Age:  ≤50 (referent) |  |  |  |
| >50 | 1.03 | 0.90-1.18 | 0.68 |
| Sex:  Male (referent) |  |  |  |
| Female | 0.98 | 0.87-1.10 | 0.71 |
| Race:  White (referent) |  |  |  |
| Non-White | 1.01 | 0.89-1.15 | 0.87 |
| Primary payer:  Private insurance (referent) |  |  |  |
| Non-Private payer | 1.04 | 0.92-1.17 | 0.56 |
| Zip code median income:  Q4 (referent) |  |  |  |
| Q1-Q3 | 1.00 | 0.87-1.15 | 0.99 |
| ECI (per count) | 1.05 | 1.00-1.11 | 0.04 |
| Hospital type:  Urban teaching (referent) |  |  |  |
| Other hospital type | 1.02 | 0.87-1.19 | 0.84 |
| Hospital region:  South/Midwest/West (referent) |  |  |  |
| North | 1.03 | 0.91-1.17 | 0.63 |
| Hospital volume:  High (referent) |  |  |  |
| Low/intermediate | 1.04 | 0.90-1.19 | 0.64 |

IRR=incidence rate ratio; LARN=laparoscopic radical nephrectomy; RARN=robotic-assisted radical nephrectomy; ORN=open radical nephrectomy; Non-White=Black, Other; Non-Private payer=Medicare, Medicaid, Other; Q1, Q2, Q3, and Q4=income quartile; ECI=Elixhauser Comorbidity Index; Other hospital type=Urban (nonteaching), Rural

| **Supplemental Table 12. Multivariable negative binomial regression analysis of LARN and RARN vs ORN for length of stay** | | | |
| --- | --- | --- | --- |
|  | Adjusted IRR | 95% CI | p-value |
| ORN (referent) |  |  |  |
| LARN | 0.80 | 0.76-0.84 | < 0.0001 |
| RARN | 0.77 | 0.73-0.80 | < 0.0001 |
| Age:  ≤50 (referent) |  |  |  |
| >50 | 0.98 | 0.93-1.04 | 0.59 |
| Sex:  Male (referent) |  |  |  |
| Female | 0.98 | 0.95-1.02 | 0.38 |
| Race:  White (referent) |  |  |  |
| Non-White | 1.04 | 0.99-1.10 | 0.08 |
| Primary payer:  Private insurance (referent) |  |  |  |
| Non-Private payer | 1.07 | 1.03-1.11 | 0.0004 |
| Zip code median income:  Q4 (referent) |  |  |  |
| Q1-Q3 | 1.02 | 0.98-1.05 | 0.37 |
| ECI (per count) | 1.05 | 1.04-1.07 | < 0.0001 |
| Hospital type:  Urban teaching (referent) |  |  |  |
| Other hospital type | 0.99 | 0.95-1.04 | 0.71 |
| Hospital region:  South/Midwest/West (referent) |  |  |  |
| North | 1.00 | 0.96-1.04 | 0.96 |
| Hospital volume:  High (referent) |  |  |  |
| Low/intermediate | 1.04 | 0.99-1.09 | 0.12 |

IRR=incidence rate ratio; LARN=laparoscopic radical nephrectomy; RARN=robotic-assisted radical nephrectomy; ORN=open radical nephrectomy; Non-White=Black, Other; Non-Private payer=Medicare, Medicaid, Other; Q1, Q2, Q3, and Q4=income quartile; ECI=Elixhauser Comorbidity Index; Other hospital type=Urban (nonteaching), Rural

| **Supplemental Table 13. Multivariable linear regression analysis of LARN and RARN vs ORN for total costs** | | | |
| --- | --- | --- | --- |
|  | e^β^ | 95% CI | p-value |
| ORN (referent) |  |  |  |
| LARN | 0.73 | 0.72-0.74 | < 0.0001 |
| RARN | 0.85 | 0.84-0.87 | < 0.0001 |
| Age:  ≤50 (referent) |  |  |  |
| >50 | 0.92 | 0.91-0.93 | < 0.0001 |
| Sex:  Male (referent) |  |  |  |
| Female | 0.94 | 0.93-0.95 | < 0.0001 |
| Race:  White (referent) |  |  |  |
| Non-White | 1.12 | 1.10-1.13 | < 0.0001 |
| Primary payer:  Private insurance (referent) |  |  |  |
| Non-Private payer | 1.08 | 1.07-1.09 | < 0.0001 |
| Zip code median income:  Q4 (referent) |  |  |  |
| Q1-Q3 | 0.95 | 0.93-0.96 | < 0.0001 |
| ECI (per count) | 1.07 | 1.07-1.08 | < 0.0001 |
| Hospital type:  Urban teaching (referent) |  |  |  |
| Other hospital type | 0.98 | 0.97-0.99 | 0.0006 |
| Hospital region:  South/Midwest/West (referent) |  |  |  |
| North | 1.17 | 1.16-1.19 | < 0.0001 |
| Hospital volume:  High (referent) |  |  |  |
| Low/intermediate | 0.97 | 0.95-0.99 | 0.001 |

e^β^=anti-log transformation of the coefficient β from regressing LN(total costs) on covariates, yielding the multiplicative difference in the expected value of total cost associated with different covariate values; LARN=laparoscopic radical nephrectomy; RARN=robotic-assisted radical nephrectomy; ORN=open radical nephrectomy; Non-White=Black, Other; Non-Private payer=Medicare, Medicaid, Other; Q1, Q2, Q3, and Q4=income quartile; ECI=Elixhauser Comorbidity Index; Other hospital type=Urban (nonteaching), Rural

| **Supplemental Table 14. Multivariable logistic regression analysis of RARN vs LARN intraoperative and postoperative outcome** | | | |
| --- | --- | --- | --- |
| Complication category | Adjusted OR | 95% CI | p-value |
| Blood transfusions | 1.10 | 0.93-1.31 | 0.28 |
| Any complications | 0.98 | 0.88-1.09 | 0.76 |
| Cardiac | 1.00 | 0.66-1.53 | 0.99 |
| Genitourinary | 0.99 | 0.79-1.25 | 0.95 |
| Respiratory | 1.37 | 1.05-1.78 | 0.02 |
| Wound/Infection | 1.07 | 0.83-1.39 | 0.60 |
| Misc. Surgical | 0.88 | 0.76-1.03 | 0.11 |
| Non-routine discharge | 1.05 | 0.95-1.15 | 0.37 |
| Death | 0.67 | 0.37-1.19 | 0.17 |

Models adjusted for patient characteristics (sex, age, race, Zip code median income, primary payer, and comorbidity index) and hospital characteristics (type, region, and volume)

LARN=laparoscopic radical nephrectomy; RARN=robotic-assisted radical nephrectomy; OR=odds ratio; CI=confidence interval

| **Supplemental Table 15. Poisson regression analysis of RARN vs LARN complication counts** | | | |
| --- | --- | --- | --- |
|  | Adjusted IRR | 95% CI | p-value |
| Complication counts | 0.99 | 0.83-1.20 | 0.97 |

Model adjusted for patient characteristics (sex, age, race, Zip code median income, primary payer, and comorbidity index) and hospital characteristics (type, region, and volume)

LARN=laparoscopic radical nephrectomy; RARN=robotic-assisted radical nephrectomy; IRR=incidence rate ratio; CI=confidence interval

**Supplemental Figure 1. Annual percentages of procedures using LARN, RARN, and ORN during 2016-2019**


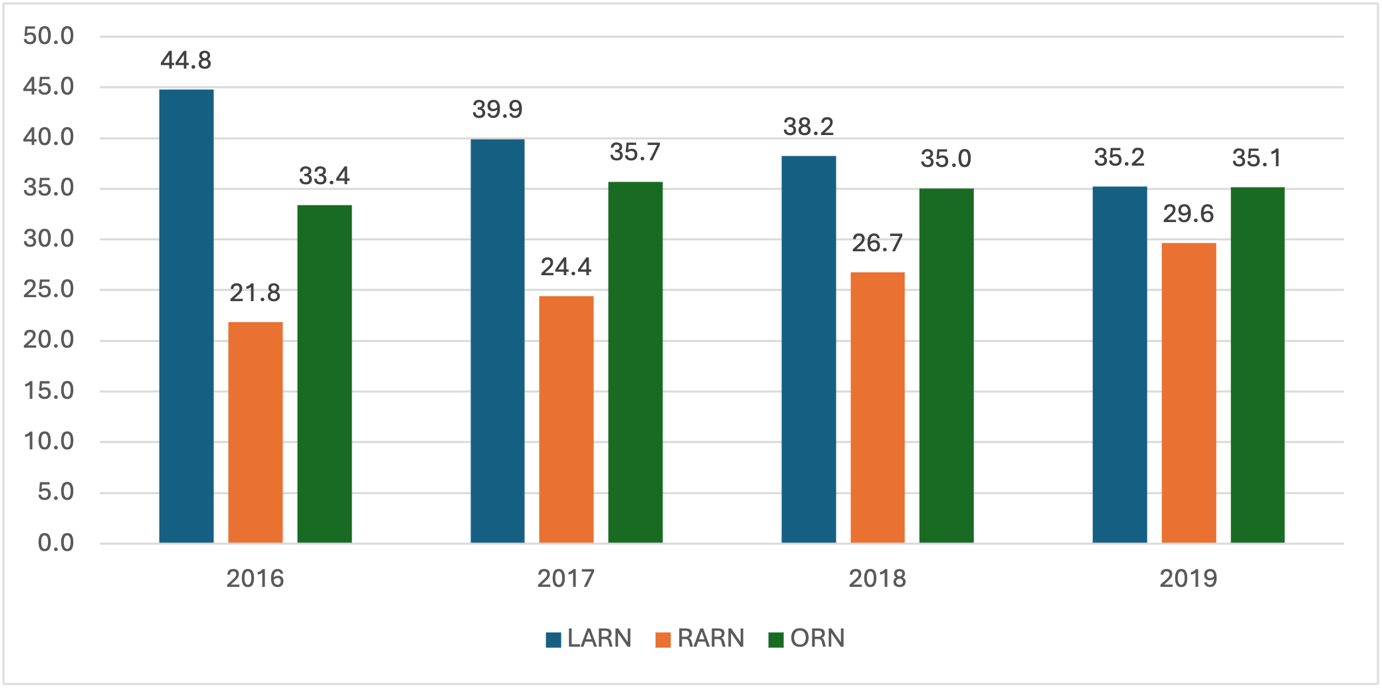

Supplement: Supplementary file 1 — Supplementary Material 1 [file 11701_2025_2995_MOESM1_ESM.docx]
